# Thresholds for identifying pathological intracranial pressure in paediatric traumatic brain injury

**DOI:** 10.1038/s41598-019-39848-1

**Published:** 2019-03-05

**Authors:** Saeed Kayhanian, Adam M. H. Young, Ross L. Ewen, Rory J. Piper, Mathew R. Guilfoyle, Joseph Donnelly, Helen M. Fernandes, Matthew Garnett, Peter Smielewski, Marek Czosnyka, Shruti Agrawal, Peter J. Hutchinson

**Affiliations:** 1 School of Clinical Medicine, University of Cambridge, Cambridge, UK; 2 Division of Academic Neurosurgery, Addenbrooke’s Hospital, University of Cambridge, Cambridge, UK; 3 Department of Paediatric Intensive Care, Addenbrooke’s Hospital, University of Cambridge, Cambridge, UK

## Abstract

Intracranial pressure (ICP) monitoring forms an integral part of the management of severe traumatic brain injury (TBI) in children. The prediction of elevated ICP from imaging is important when deciding on whether to implement invasive ICP monitoring for a patient. However, the radiological markers of pathologically elevated ICP have not been specifically validated in paediatric studies. Here in, we describe an objective, non-invasive, quantitative method of stratifying which patients are likely to require invasive monitoring. A retrospective review of patients admitted to Cambridge University Hospital’s Paediatric Intensive Care Unit between January 2009 and December 2016 with a TBI requiring invasive neurosurgical monitoring was performed. Radiological biomarkers of TBI (basal cistern volume, ventricular volume, volume of extra-axial haematomas) from CT scans were measured and correlated with epochs of continuous high frequency variables of pressure monitoring around the time of imaging. 38 patients were identified. Basal cistern volume was found to correlate significantly with opening ICP (r = −0.53, p < 0.001). The optimal threshold of basal cistern volume for predicting high ICP ($\ge$20 mmHg) was a relative volume of 0.0055 (sensitivity 79%, specificity 80%). Ventricular volume and extra-axial haematoma volume did not correlate significantly with opening ICP. Our results show that the features of pathologically elevated ICP in children may differ considerably from those validated in adults. The development of quantitative parameters can help to predict which patients would most benefit from invasive neurosurgical monitoring and we present a novel radiological threshold for this.

## Introduction

Traumatic brain injury (TBI) remains a major cause of death and morbidity worldwide^1^. It is a particular public health concern in the paediatric population, since the vast majority of TBI occurs in children and young adults^2^. The clinical management of TBI is centred around ensuring adequate cerebral perfusion in order to limit secondary brain injury^3^. Adequately controlled intracranial pressure (ICP) is a key determinant of cerebral perfusion and the decision for surgical, or the most aggressive medical, interventions are guided by ICP-thresholds. The gold-standard for monitoring ICP is through the insertion of an invasive ICP monitor or external ventricular drain. However, these procedures carry risks of haemorrhage, infection, and seizures^4–6^. A clinical assessment, using the Glasgow coma score, to assess the severity of TBI, is frequently complemented by radiological markers, notably features seen on computerized tomography (CT) brain imaging, to guide the requirement for ICP-monitoring.

Radiological features that correlate elevated ICP (e.g. midline shift, basal cistern, and sulcal effacement) have been studied in adults and correlated to long-term outcomes, as demonstrated by Rotterdam and Marshall scoring systems^7,8^. However, these features have not been well validated in paediatric cohorts and there is increasing recognition that there may be clinically significant differences between adult and paediatric CT-head features of raised ICP. Notably, compressed or obliterated basal cisterns signify raised ICP in adults, and correlate to poor outcomes, but in paediatric cohorts Kouvarellis *et al*.^9^ demonstrated elevated ICP cannot be excluded even when patent basal cisterns are observed^9,10^. Moreover, the type and severity of the injuries visible on CT images has been shown to differ significantly between adults and paediatric cohorts with the same Glasgow Coma Score (GCS) after TBI^11^.

These reported differences in radiological features may be explained by the known differences in anatomy, biomechanics and pathophysiology of paediatric head injury versus that in adults. Children are known to have a larger head to body size ratio, thinner cranial bones, and less myelinated neural tissue^12^. This not only makes TBI more likely but may also allow for a greater degree of accommodation of increased ICP before exhibiting clinical signs. Compared with adults, children also exhibit a greater propensity to developing diffuse brain swelling after TBI, which has been variously postulated to be because of immature or impaired autoregulation of cerebral blood flow, an enhanced inflammatory response, and increased blood-brain barrier permeability in the developing brain^13–15^.

There is a need for specifically validated paediatric models of elevated ICP features on CT imaging. Moreover, a recent randomised control trial in adults has questioned whether, in current practice, invasive monitoring is over-utilised^16^. It would, therefore, be desirable to develop quantified predictive parameters, which could be consistently applied, to identify the cohort of paediatric patients who are most at risk of elevated ICP.

In this retrospective analysis of children with TBI, we report the correlations between the features of referred CT scans with the opening ICP at the time of surgery.

## Methods

### Patients

The clinical records of patients admitted to Cambridge University Hospital’s (CUH) Paediatric Intensive Care Unit (PICU) between January 2009 and December 2016 were retrospectively reviewed. Patients aged 16 and under, who had sustained a severe traumatic brain injury and had an intracranial pressure monitor (Codman®) inserted as part of their clinical care, were included. The analysis of data within this study for the purposes of service evaluation was approved by CUH Audit and Evaluation Department (Ref: 2143) and did not require ethical approval or patient consent.

All patients received full active management of their TBI.

### CT image acquisition

CT images were obtained from CUH Picture Archiving and Communication System database. All CT scans were performed with a 16-section multi-detector row CT scanner (Somatom Sensation 16 scanner, Siemens, Germany). The first CT scan performed at CUH for each patient was used for the measurements.

### CT image analysis

**Figure 1:**
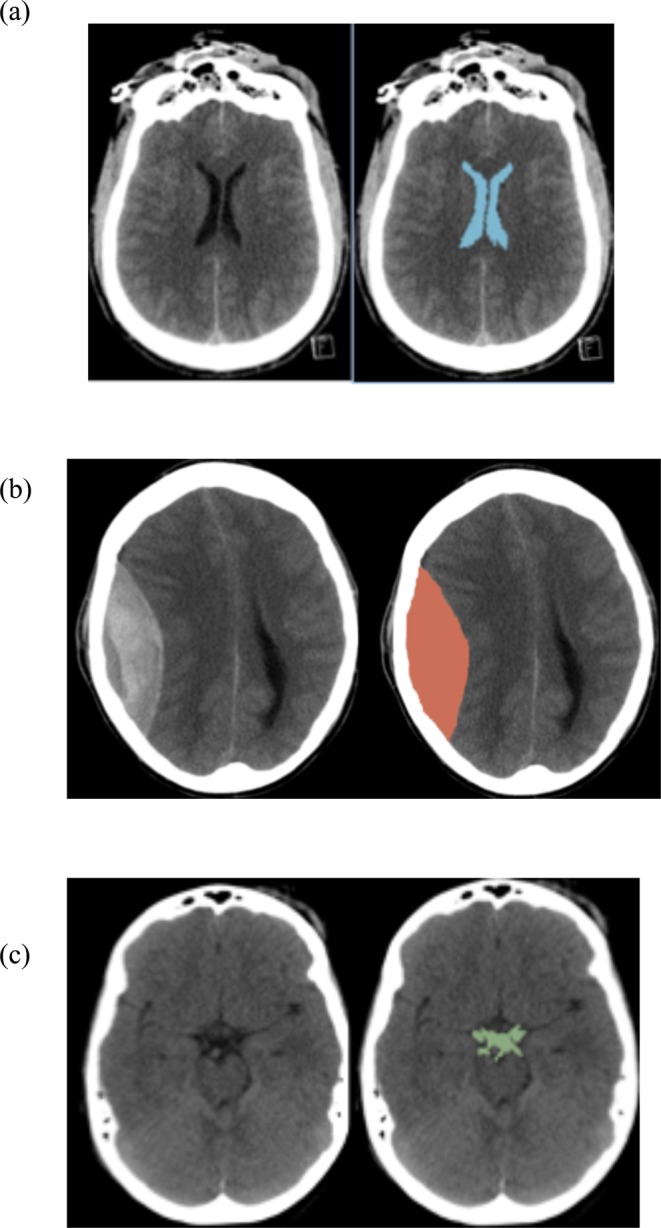
Examples of semi-automated measurement. Volumes of interest for each patient were segmented for each slice of the scan and then summed together. Representative examples for: (**a**) segmentation of the lateral ventricles (**b**) segmentation of an extra-axial haematoma (**c**) segmentation of the suprasellar cistern (analysis included all intracranial subarachnoid cisterns).

Two investigators (S.K., R.E.), who were blinded to patient outcomes, used manual segmentation to quantify the following volumes-of-interest (VOI) using semi-automatic software (3D Slicer, Boston, MA, USA): intracranial volume (ICV), ventricular system, extra-axial haematoma, and basal cisterns. The features of interest on each slice of a patient scan were manually segmented and then summed by the software to calculate a total volume (Fig. 1). Ventricular system measurements were the sum of the volumes of third, fourth, and lateral ventricles. The basal cisterns volume included all of the intracranial subarachnoid cisterns.

### Statistical analysis

Each VOI was corrected for ICV (VOI/ICV). Spearman’s coefficient was used to evaluate the correlation between the VOIs and opening ICP at the time of invasive monitor insertion. The significance level was set to 0.01 to accommodate high frequency of data points. The performance of different VOIs in predicting high ICP (defined as $\ge$20 mmHg) was examined by constructing receiver-operating characteristic curves of compartmental volume against dichotomised (high or normal) opening ICP. The area-under-the-curve (AUC) was then calculated, with AUC > 0.8 generally considered to indicate good performance. Delong method was used to calculate the 95% confidence interval. Youlden’s J-statistic was used to calculate the optimal threshold of basal cisterns volume, balancing sensitivity and specificity for predicting high ICP.

## Results

### Demographics

38 patients with a mean age of 9.4 years (range 3–16) were admitted to Cambridge University Hospital Paediatric Intensive Care Unit (PICU) with a TBI and required invasive neurosurgical monitoring (Table 1). Twenty-eight (74%) were alive at 6 months and 25 (66%) were deemed to have a favourable outcome. Thirty patients (79%) were deemed to have an isolated head injury with the remaining having sustained poly-trauma. The incidence of poly-trauma did not have an impact on the outcome. All ICP monitors were inserted after a clinical examination determined poor neurology (GCS < M5), requiring intensive medical management. This was usually within 6 h of injury. No patients were excluded on the basis of the timing of ICP insertion. Prior to the injury one child had mild learning disabilities, and one had attention deficit hyperactivity disorder. All patients were maintained at normothermia. Four patients had external ventricular drain inserted. Two patients had a decompressive craniectomy. 79% of patients had vassopressor/inotrope support. 58% of patients had insulin infusions in an attempt to control glucose levels.

**Table 1:** Demographic data of paediatric cohort.

	Survived (n = 28)	Non-survivors (n = 10)	p value
Age, mean ± SD	8.8 + 2.8	11.2 + 5.2	0.10
Male (%)	21 (75)	7 (70)	0.72
Admission GCS, median (range)	9 (3–9)	3 (3–9)	0.03
Motor Score	6 (1–6)	1 (1–5)	0.02
**Pupils**			
*Reactive (%)*	92	30	0.02
*Fixed Unilaterally (%)*	4	20	0.03
*Fixed Bilaterally (%)*	4	50	0.01
Hypoxia	7	33	0.40
Hypotension	7	20	0.08
Initial ICP, mean ± SD	14.6 + 6.4	20.7 + 7.2	0.01
Initial PRx, mean ± SD	−0.04 ± 0.16	0.08 + 0.43	0.01

Physiologic monitoring values from the beginning of monitoring are shown in Table 1. The mean (SD) ICP was 16.2 (8.0) mmHg, MAP was 86.4 (9.2) mmHg and PRx was 0.05 (0.18) a.u.

### Intracranial measurements and ICP

The Modified Marshall scores ranged from 2–5 with a mode of 3.

#### Basal cisterns volume and ICP

**Figure 2:**
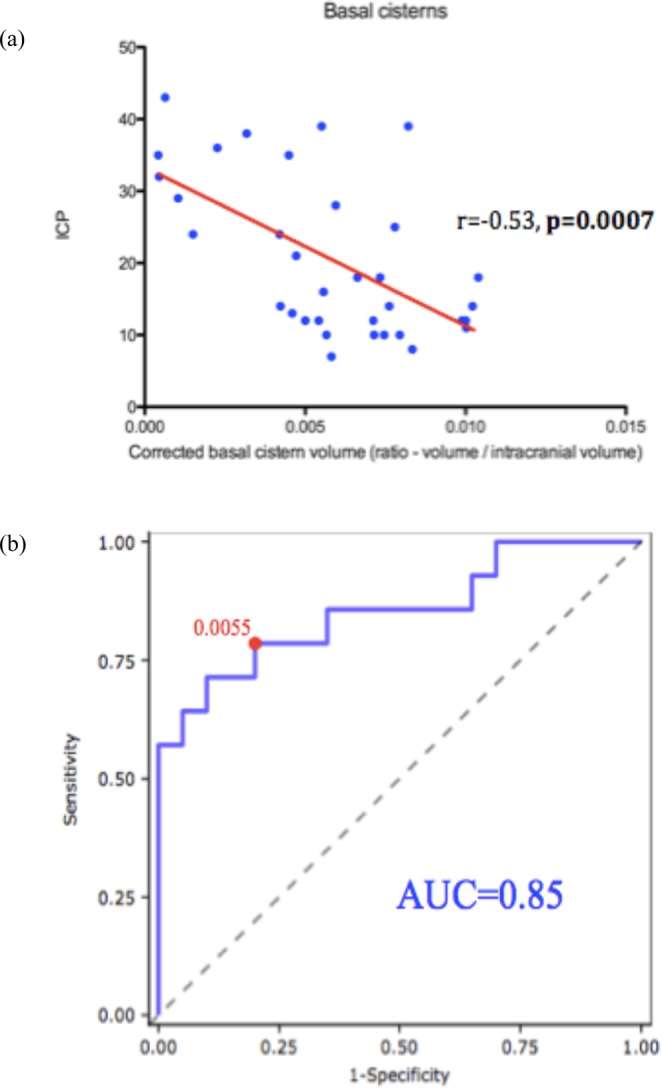
Basal cistern measurements and association with ICP. (**a**) Linear regression of corrected basal cistern volume found a significant correlation with initial ICP (r = −0.53, P < 0.001). (**b**) Assessment of relative volume of basal cistern measurements. The area under Receiver-operating characteristic (ROC) curve was 0.85.

The average basal cistern volume of the cohort was 7.45 ml. The mean corrected ratio of basal cistern volume in the cohort was 0.00596. Linear regression of corrected basal cistern volume found a significant correlation with opening ICP (r = −0.53, p < 0.001) (Fig. 2a). The optimal threshold of basal cistern volume for predicting high ICP ($\ge$20 mmHg), identified by maximising the J-statistic, was a relative volume of 0.0055 (sensitivity 79%, specificity 80%). The area under Receiver-operating characteristic (ROC) curve was 0.85 (0.71–0.99 CI; Fig. 2b).

#### Ventricular volume and ICP

**Figure 3:**
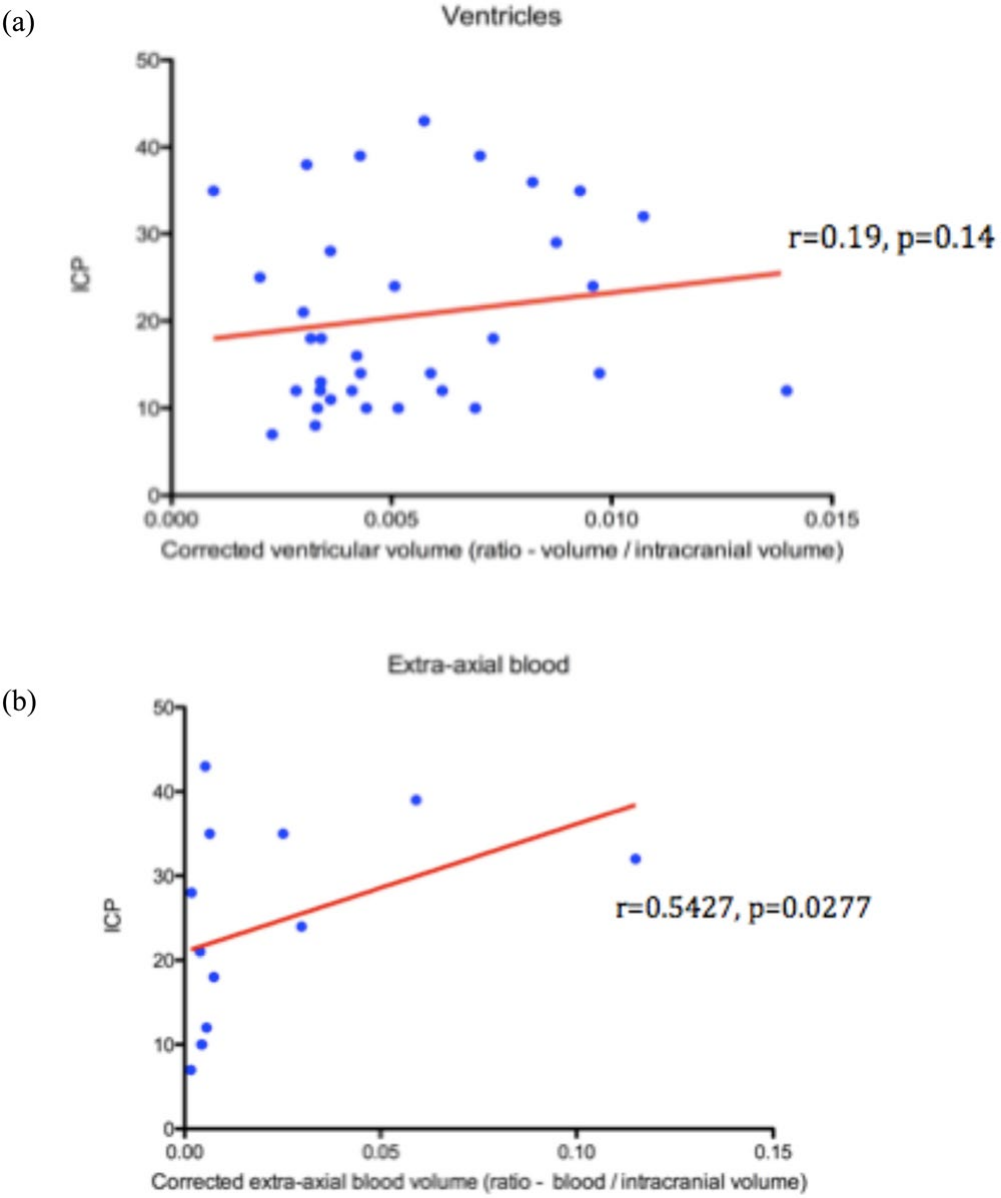
Ventricular and extra-axial blood associations with ICP. (**a**) The mean corrected ratio of ventricular volume in the cohort was 0.00552. There was no significant correlation between ventricular volume and initial ICP (r = 0.19, p = 0.14). (**b**) The mean corrected ratio of extra-axial blood volume, in the sub-cohort (n = 13) demonstrating this feature, was 0.0207. No significant correlation was noted between corrected extra-axial blood volume and initial ICP (r = 0.54, P = 0.03).

The average ventricular volume within the cohort was 10.52 ml. The mean corrected ratio of ventricular volume in the cohort was 0.00552. There was no significant correlation between ventricular volume and opening ICP (r = 0.19, p = 0.14) (Fig. 3a). Linear regression of corrected ventricular volume and relative volume also failed to find a significant correlation with mean ICP over the initial 5-day period. However, ventricular volume was also not significantly related to the mean number of pathological ICP plateau waves detected over the initial 5-day period.

#### Extra-axial blood and ICP

The average volume of extra-axial blood was 19.76 ml. The mean corrected ratio of extra-axial blood volume, in the sub-cohort (n = 13) demonstrating this feature, was 0.0207. No significant correlation was noted between corrected extra-axial blood volume and opening ICP (r = 0.54, p = 0.03) (Fig. 3b). It was also examined whether the presence of extra-axial blood impacted the ICP over the initial 5-day period following injury or the number of plateau waves during this period. Although extra-axial blood was removed in these cases, correlation would usually exist with refractory swelling in such patients. In this cohort, it was found that there was no correlation.

## Discussion

Invasive monitoring of ICP after severe TBI is widely implemented as standard care. The most recent guidelines for medical management of severe TBI include many treatments that are predicated on having continuous ICP monitoring data for a patient^3^. However, much of the evidence for this practice in children has been extrapolated from studies in adult cohorts^17^.

The significance of pathologically elevated ICP in children as a prognostic marker of poor outcomes has been previously demonstrated^18^. Our results demonstrate that patients with raised intracranial pressure can be predicted with significant accuracy using our model and as such these patients may benefit from ICP monitor placement.

The data from this retrospective study suggests basal cisterns volume as the only quantifiable CT-parameter that correlates significantly with initial ICP in a paediatric cohort. This is reassuring, given that effacement of the cisterns at the level of the midbrain is considered to be the most widely used radiological estimate for the degree of brain swelling^9^.

It is notable that ventricular volume was not shown to correlate with ICP in children, unlike in adults cohorts where ventricular compression has been shown to be predictive of elevated ICP^10,19^. This finding may be partly explained by the fact that adults have a much lower ratio of brain volume/cerebrospinal fluid (CSF) volume, so brain swelling escalating to measurable ventricular compression in adult brains may suggest a more severe injury than when it occurs in children^20^.

It is interesting also that extra-axial blood volume was not significantly correlated with ICP, with midline shift (a surrogate marker of extra-axial blood volume) being previously demonstrated as associated with elevated ICP in a mixed adult and adolescent cohort^21^. We highlight, however, the small subset (n = 13) of our cohort with such a radiological feature.

**Figure 4:**
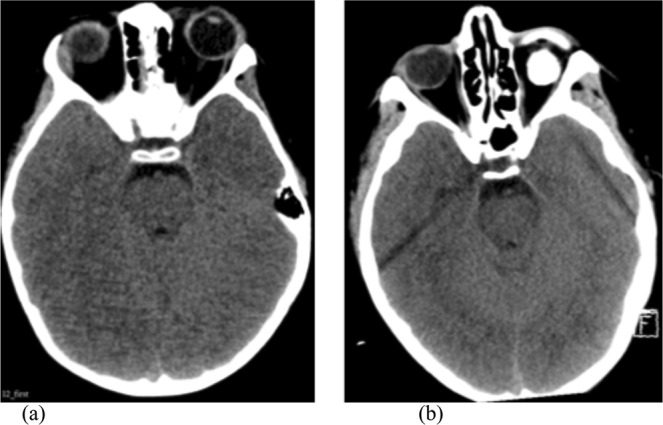
Representative CT images of basal cistern appearance. Patient with a corrected basal cistern volume ratios of: (**a**) 0.0054 [just below threshold], ICP = 12 mmHg (**b**) 0.0058 [just above threshold], ICP = 25 mmHg. Both demonstrate patent suprasellar cisterns.

We also demonstrate threshold measurements of the basal cisterns that are predictive of high ICP in children. This threshold supports Korvellis *et al*.’s previous findings, with the appearance of the basal cisterns at this cut-off being unambiguously patent (Fig. 4), despite corresponding to a pathologically raised ICP. This further emphasises the necessity for caution in ascribing a CT scan from a paediatric TBI patient as ‘normal’ simply because of open basal cisterns, as might otherwise be appropriate in adult patients.

This study is limited by a small sample size and the demonstrated thresholds would need to be validated in a larger cohort before they could be of use in clinical practice. Moreover, this study looked retrospectively at children with the most severe TBIs (since the patients all required invasive monitoring). In order to be of clinical value, these thresholds would need to be reliable when applied prospectively to a mixed cohort of severe and moderate TBI, which has not been demonstrated here.

We highlight also that the measurements in this study were made using semi-automatic software, requiring manual segmentation for the compartments of interest. This method is time-consuming and, as such, inappropriate for time-critical clinical settings. Many studies have attempted to develop fully automated software for the volumetric analysis of CT head scans, though these have been limited to one or two compartments of interest^22–24^. A fully-automated and accurate volume analysis software would need to be realised before threshold-analysis could be effectively utilised as a clinical decision-making tool in the context of TBI.

However, we hope this study will encourage further work to develop quantitative thresholds and further attempts to automate volumetric analysis of CT head imaging. These thresholds would provide useful guidance as to when invasive ICP-monitoring would be appropriate and allow reassurance when patients are not suitable for monitoring (e.g. coagulopathies) or, in the global context of neurosurgery, when invasive monitoring is not available. Indeed, the requirement for the expertise to insert such a device can result in delays in the implementation of guided medical therapy: some areas of the world are served only by 1 neurosurgeon per 9 million patients (compared with the 1 per 80,000 in developed countries)^25^.

## Conclusion

Our results provide further evidence for the marked differences in radiological appearance of elevated ICP in children compared to adults. We demonstrate a novel quantified threshold of basal cistern volume that is predictive of pathologically elevated ICP.
